# The Prevalence and Health Impacts of Frequent Work Discrimination and Harassment among Women Firefighters in the US Fire Service

**DOI:** 10.1155/2019/6740207

**Published:** 2019-03-20

**Authors:** Sara A. Jahnke, Christopher K. Haddock, Nattinee Jitnarin, Christopher M. Kaipust, Brittany S. Hollerbach, Walker S. C. Poston

**Affiliations:** Institute for Biobehavioral Health Research, National Development & Research Institutes, 1920 W. 143rd Street, Suite 120, Leawood, KS 66224, USA

## Abstract

**Intro:**

Both discrimination and harassment directly impact mental and physical health. Further, workplace discrimination degrades workplace culture and negatively impacts health behaviors, job-related outcomes, and family dynamics. Women represent a small proportion of the fire service and are often the targets of discrimination/harassment, yet little research documents the impact of such experiences. The purpose of this study was to evaluate the relationship between chronic work discrimination and/or harassment and women firefighters' (FFs) physical and mental health, substance abuse, and job efficacy, stress, and satisfaction.

**Methods:**

Snowball sampling was used to solicit participation from women career FFs. Participants completed an online survey regarding physical and mental health, health behavior, job efficacy/stress/satisfaction, and family well-being. Logistic regression examined the impact of work discrimination-harassment severity on dichotomous variables.

**Results:**

1,773 had complete data on their experiences with work-related discrimination and harassment. Women reported experiencing verbal (37.5%) and written (12.9%) harassment, hazing (16.9%), sexual advances (37.4%), and assaults (5.1%) in the fire service. FFs in the highest tertile of work discrimination-harassment severity reported over 40% more poor health days in the last 30 days (OR=1.42; 95%CI=1.33-1.51; p<0.001). Women who experienced moderate and severe discrimination/harassment had negative mental health outcomes including higher prevalence of depressive symptoms, anxiety, and PTSD symptoms. Those who experienced high rates of discrimination and/or harassment also were more likely to report issues with alcohol consumption.

**Conclusion:**

The impact of discrimination and harassment, related negative physical and mental outcomes, low levels of job satisfaction, and negative impact of these experiences on family/home stress likely take a significant toll on women in the fire service. Findings confirm and extend previous work suggesting there is a need to improve the mental and physical health of women FFs. Future work should examine the prospective relationship between discrimination/harassment and poor health outcomes and potential policies/practices to reduce these negative behaviors.

## 1. Introduction

Both discrimination and harassment in the workplace, their correlates, and outcomes have been receiving an increasing amount of attention in the literature [1–8]. Discrimination occurs when an individual or organization set unfair conditions that impair the ability of another person(s) to successfully conduct their work typically based on a specific attribute such as gender and race/ethnicity [2]. Harassment, on the other hand, occurs when negative actions are taken specifically against an individual because of their status in a protected group [2]. While general harassment can be discriminatory, people can experience discrimination without being harassed. The current work focuses on both constructs as issues of workplace mistreatment among female firefighters.

There is a large body of epidemiologic literature, spanning over several decades, demonstrating that both racial and gender-based discrimination and harassment are associated with negative impacts on mental and physical health and job-related outcomes [2, 5–9]. Work in this area is occurring worldwide [10, 11]. While studies vary considerably with respect to how they measure discrimination and/or assess health, most consistently find associations between discrimination and/or harassment and a variety of mental and physical health outcomes (e.g., anxiety, depression, posttraumatic stress, self-rated health, overall stress response, blood pressure, cardiovascular disease, diabetes, somatic symptoms, and a number of medical conditions) [1, 2, 4, 5, 7, 8] and health behaviors (e.g., physical activity, exercise, diet, alcohol, tobacco, and other substance use) [3, 7, 8]. Various investigators have posited models explaining the impact of both discrimination and harassment on health with many suggesting that experiencing either can affect mental and physical health by impacting health behaviors that influence health outcomes and/or by increasing affected individuals' stress responses, which in turn affects health outcomes [2, 6–9].

The specific effects of work-related discrimination and harassment on health have received some attention in the peer-reviewed literature [6–8]. Okechukwu and colleagues [1] synthesized the literature on the impacts of workplace discrimination, harassment, and bullying on physical and mental health, health behaviors, job-related outcomes, and family. They proposed a conceptual model for how workplace injustices, i.e., discrimination, harassment, and bullying, can impact mental and physical health, health behaviors, and job- and family-related outcomes either directly or through other factors, such as differential exposures to occupational hazards, stress, and the influences of each of the outcome domains on one another (changes in health behaviors can impact mental and physical health, poor physical or mental health can affect job-related outcomes and family functioning, etc.). They concluded that a growing body of evidence supports the premise that workplace discrimination and harassment affect all of the outcome domains (i.e., mental and physical health, health behaviors, job-related outcomes, and family well-being) proposed in their model [1].

Women firefighters are substantially underrepresented (i.e., between 3.0-5.1% [12]) in the US fire service. In fact, among tactical professions, which also includes law enforcement and the military, the proportion of women is the lowest in the fire service. It is even lower than occupational groups like the US Marine Corps, where most job classifications involve potential exposure to combat and women were legally excluded from combat roles until 2013 [9].

Over the past 20+ years, a number of studies of women firefighters have documented troubling gender-based discrimination and harassment, although not specifically focused on the health impacts [13–18]. In the earliest studies, Yoder and colleagues [15, 16, 19] used mixed-methods studies (i.e., structured interviews and surveys) with small samples of African American (N=24) and White (N=24) women firefighters. In this series of studies, African American women firefighters reported high rates of unwanted sexual teasing and jokes, letters, notes, calls, and looks and most reported feeling as if they had been sexually harassed at some time during their firefighting career [16, 19]. Both African American and White women firefighters reported pervasive problems with being excluded from the fire service culture, usually through being provided insufficient instruction and support, being micromanaged, and feeling hostility from their colleagues about their presence in the fire service [16, 19].

More recently, Hulett and associates [13] completed “A national report card on women in firefighting” in which they surveyed 457 women and 218 men firefighters across 48 states in the US. As well, they collected data from 114 fire departments across 39 states. In their survey, the vast majority of women firefighters (84.7%) reported experiencing different treatment based on gender. Disturbingly high proportions of women firefighters also reported high levels of shunning/isolation (50.8%), verbal harassment (42.9%), sexual advances (30.2%), and assault (6.3%).

Griffith and colleagues [17] surveyed 339 women firefighters using an Internet-based survey about their perceptions of their careers in the fire service. While the survey was not specifically focused on discrimination, more than half of the respondents (54%) indicated they did not feel they were treated as equals by their male colleagues. Griffith and colleagues [18] conducted another Internet-based survey study about perceptions of bullying in the fire service in a sample of 113 firefighters (50% women) and reported again that the majority of women firefighters believed they were treated differently based on gender (79%), but only a small proportion of men felt that way (14%; p<0.001). Women firefighters also were more likely to express the opinion that (1) supervisors did not address concerns about gender-related incidents (32% vs. 3%, p<0.001, for women vs. men, resp.); (2) gender is a barrier to career development (44% vs. 5%, p<0.001, for women vs. men, resp.); and (3) promotions are not decided fairly (41% vs. 16%, p=0.001, for women vs. men, resp.).

It is possible that discrimination and harassment increase the challenges of recruiting and retaining women firefighters, which likely has contributed in their very low numbers in fire service [13, 17, 18]. In addition to the impact of discrimination and harassment on representation, it is likely that it also negatively impacts the health of women firefighters. Rosell and colleagues [20] conducted a survey of 206 women firefighters. Women firefighters who had experienced sexual harassment were significantly more likely to report job stress (80% vs. 61%; p<0.001) and using sick leave to avoid work (29% vs. 14%; p=0.020) when compared to those who did not report being harassed. More recently, Boffa and colleagues [21] reported the results of a web-based cross-sectional survey examining correlates of suicidality and psychopathology among firefighters. In this substudy, they had 290 current women firefighters of which 22% reported a history of sexual harassment. When compared to those with no history of sexual harassment, women firefighters reporting a history of sexual harassment were significantly more likely to have experienced suicidal ideations, anxiety, depression, and insomnia symptoms and were at greater risk for PTSD.

The purpose of this study is to evaluate the impact of chronic work discrimination and harassment on women firefighters' physical and mental health, substance abuse, and job efficacy, stress, and satisfaction. While the parent study from which these data are drawn was not specifically designed to examine all forms of discrimination and harassment (i.e., the parent study was designed to survey a broad range of health domains with a focus on reproductive health concerns), we used a well-established measure of chronic work discrimination-harassment over the past year and examined its association with a number of important health outcomes that are relevant and informative.

This study fills a critically important gap in the scientific literature as there have only been a few numbers of studies with small-medium sized samples documenting the experiences of discrimination and harassment faced by women firefighters [13–19, 22]. In addition, there are only three quantitative survey studies that examined how experiences with sexual harassment or general harassment affected a limited number of health outcomes (e.g., job stress appraisals, reported use of sick days, suicidality, and psychopathology) [20–22]. This study aims to move the field forward by considering diverse forms of mistreatment in firefighters, the relationships between them, and stress-related processes and their outcomes. Therefore, this study offers a more comprehensive picture of discrimination and harassment of women firefighters, the profession with the lowest women proportion or ratio in the US. Documenting the occurrence, frequency, and severity of chronic work discrimination and harassment and its impact on the health of women firefighters is the first critical step to understanding how these factors affect recruitment and retention and addressing the low numbers of women in the US fire service.

## 2. Methods

### 2.1. Sampling Methods

Women firefighters can arguably be described as a “hidden population” because of their extremely low representation (i.e., ≈ 5%) in the US fire service [9] and because, as mentioned in previous research, no central registry of firefighters currently exists. Thus, there is no national registry of firefighters, or women firefighters in particular, that can be used to derive a sampling frame.

Recruitment strategies are more specifically outlined in previous publications [23, 24] but included recruitment through contacts with previous participants, emails from organizations (e.g., iWomen, International Association of Firefighters), listserves (e.g., www.firefighterclosecalls.com), and through social media postings. Secondary recruitment included requesting any women who completed the survey to share the solicitation with their women colleagues. All women firefighters interested in partaking in the study were directed to a web-based survey.

### 2.2. Internet Survey Protocol

This study and its protocols were approved by the Institutional Review Board (IRB) of the National Development and Research Institutes, Inc. Details about the survey protocol and consent can be found in Jahnke et al. [23] and Haddock and colleagues [24]. This survey focused on women in the career fire service specifically rather than including volunteers as career firefighters are exposed to the greatest risk, are more active responding to calls, and spend more time in the culture of the firehouse [24].

Participants were directed to the online survey that first presented an informed consent document. By clicking the link, they confirmed their consent to participate. In total, 1,773 women had complete data on their experiences with work-related discrimination and harassment. Participants were primarily from the US (98.0%) with most of the remaining residing in Canada.  Table 1 contains descriptive data about the sample.

### 2.3. Measures

Standard individual demographics (e.g., age, race/ethnicity) and occupational history (e.g., current rank and position, years in the fire service) were collected.

#### 2.3.1. Exposure

The* Chronic Work Discrimination and Harassment: Abbreviated* (CWDH-A) Scale, which was adapted from the Perceived Racism Scale for use in the Chicago Community Adult Health Study [10, 25–28], measures the occurrence and frequency of perceived chronic interpersonal discrimination that individuals experience at work. Women firefighters were provided an introduction that said “Here are some situations that can arise at work. How often have you experienced them in the past 12 months?” and then asked to report how often the following occurred (i.e., never, less than once a year, a few times a year, a few times a month, and at least once a week): (1) How often do you feel that you have to work twice as hard as others to get the same treatment or evaluation? (2) How often are you watched more closely than other workers? (3) How often are you unfairly humiliated in front of others at work? (4) How often do your supervisor or coworkers make slurs or jokes about racial or ethnic groups? (5) How often do your supervisor or coworkers make slurs or jokes about women? and (6) How often do your supervisor or coworkers make slurs or jokes about gays or lesbians?

Questions 1–3 are generally viewed as measuring experienced discrimination while 4-6 are viewed as measuring environments that allow harassment [27, 28]. Both scales have published reliability (Cronbach's alpha for discrimination = 0.73 and harassment = 0.76-0.84) [27, 28]. Reliability in our sample was alpha = 0.825, 0.908, and 0.841 for the discrimination, harassment, and composite scales, respectively. Similar to previous studies using perceived discrimination scales and the CWDH-A specifically [10, 27], we scored the frequency of discrimination and harassment items on a scale of 1-5 and summed firefighters' responses to the six questions. Thus, the potential range of scores was between 6 (no discrimination or harassment) to 30 (high discrimination and harassment), with higher scores indicating greater experiences with work discrimination and harassment.

*Gender Based Harassment.* We also asked women firefighters about whether or not they ever had experienced the following types of harassment because of their gender using questions from National Report Card on Women in Firefighting by Hulett and colleagues [13]: (1) verbal harassment; (2) written harassment (e.g., notes, cartoon, other written materials); (3) hazing; (4) sexual advances; and (5) assault.

### 2.4. Outcomes

Our outcomes assessments were modeled on the conceptual framework similar to that provided by Okechukwu and associates [1], which suggests that work discrimination-harassment can affect the following outcomes: (1) physical health; (2) mental health; (3) health behaviors; (4) job-related factors such as satisfaction, stress, advancement, and performance; and (5) family well-being. Thus, we assessed outcomes in each of these broad domains.

(*1) Physical Health (Obesity, Poor Physical Health Days, and Injury).* Self-reported height and weight were used to compute body mass index (BMI; kg/m^2^) and obesity status (BMI≥30kg/m^2^). Self-reported weight and height, and BMI estimates derived from them, are highly correlated with their respective measured values in US firefighters [29]. The number of poor physical health days during the last 30 days was assessed using a question from the CDC Behavioral Risk Factor Surveillance System (BRFSS): “Now thinking about your health, which includes physical illness and injury, for how many days during the past 30 days was your physical health not good?” [30–32]. This question has established reliability and validity [32], is predictive of important longitudinal health care utilization and outcome variables such as physician visits, hospitalizations, and mortality, and is used as part of an overall health rating system for the US [33, 34]. In addition, it is a documented health disparity among minority firefighters [35, 36]. Finally, women firefighters were asked whether they had experienced an occupational injury in the past 12 months based on a standard item developed for use in the fire service [37–39].

(*2) Mental Health.* Current depression was measured using the Center for Epidemiological Studies Short Depression Scale (CES-D10 [40]). The CES-D10 includes questions about the frequency of both feelings and behaviors during the past week. The CES-D10 has been found to be highly reliable among the general population (Spearman-Brown, split halves r=0.85) and in patient samples (r=0.90 [40]). A score of 4 or more is indicative of potential clinical depression.

The Mental Health Inventory Anxiety subscale (MHI-A [41]) was used to assess anxiety. The MHI-A subscale measures current (past month) symptoms of anxiety and is a widely accepted measure that has been used in a number of studies, including the RAND Health Insurance Experiment as part of an overall tool to assess psychological well-being and distress [41–43]. The MHI-A has a score range of 9-54 with higher scores indicating higher levels of anxiety. The MHI-A has demonstrated reliability (Cronbach's alpha=0.90; one-year stability correlation=0.63) and validity when compared with other measures of psychological distress [41–43].

Symptoms of trauma were assessed with the Trauma Screening Questionnaire (TSQ). The TSQ is a brief screening instrument that consists of 10 symptom-based questions that were experienced over the past week including intrusive thoughts, upsetting dreams, reliving of the experience, physical responses (e.g., fast heartbeat, churning stomach), sleep disturbances, irritability or angry outburst, difficulty with concentration, heightened awareness, and feeling jumpy or easily startled. A score of 6 or more positive responses suggests potential PTSD [44].

(*3) Health Behaviors (Substance Use and Physical Activity).* Problem drinking patterns and tobacco use were assessed using standard approaches that also have been successfully implemented firefighter substance use epidemiological studies [24, 45, 46]. Problem drinking behaviors were measured by the CAGE questionnaire [47–49]. The CAGE asks the following questions: (1) Have you ever felt you should cut down on your drinking? (2) Have people annoyed you by criticizing your drinking? (3) Have you ever felt guilty about your drinking? and (4) Have you ever had a drink first thing in the morning to steady your nerves or get rid of a hangover (eye-opener)? Those responding positively to two or more of the questions are considered at risk for problem drinking [48, 49].

Binge drinking was assessed with the item: “Considering all types of alcoholic beverages, how many times during the past 30 days did you have 4 drinks or more on an occasion?” [46, 47]. Driving while intoxicated was measured with the following item: “During the past 30 days, did you drive a car or other vehicle on any occasion when you perhaps had too much to drink?” Participants responded either “Yes” or “No” [47].

Current smokers were those who responded positively to the standard tobacco surveillance questions: (1) Have you ever smoked a cigarette, even just a puff? (2) Have you smoked at least 100 cigarettes in your entire life? [Note: 5 packs = 100 cigarettes]; and (3) Have you smoked a cigarette, even just a puff, in the past 30 days? Current smokeless tobacco users were participants who acknowledged using chewing tobacco, snuff, or dip in the last 30 days [45, 50].

Physical activity level was assessed using the Self-Report of Physical Activity (SRPA) Questionnaire [51–53]. The SRPA provides a global, physical activity self-rating during the last 30 days. Participants were asked to indicate their level of fitness on a scale of 0 (sedentary) to 7 (3 or more hours of vigorous activity per week). The SPRA's validity compared to maximal oxygen consumption has been established [51–53].

(*4) Job Efficacy/Stress/Satisfaction.* Firefighter Self-Efficacy was measured using “The Firefighter Coping Self-Efficacy Scale” (FFCSE [54]). The FFCSE measures firefighters' perceptions of self-confidence in managing on-the-job stressful and traumatic experiences. High internal consistency has been reported (e.g., Cronbach's alpha = 0.90 - 0.92). The FFCSE exhibited strong concurrent validity with measures of work-related stress, posttraumatic stress symptoms, general well-being, and social support.

Job stress was assessed using the following questions [55]: (1) During the* past 12 months*, how much stress did you experience* at work* while carrying out your duties in the fire service? and (2) During the* past 12 months*, how much did stress* at work* interfere with your ability to perform your duties in the fire service? Job satisfaction and organizational commitment were assessed based on the following items from previous studies [12, 35]: (1) “I am optimistic about my future success with this fire department”; (2) “I am satisfied with my job at the fire department”; (3) “I am satisfied with the morale of the people I work with in the fire service”; (4) “I am satisfied with the morale of the fire department”; and (5) “My work in the fire department gives me a sense of accomplishment.” Response options were a five-point Likert scale ranging from “Very much disagree” to “Very much agree” and scored in a continuous fashion, consistent with similar scales [56].

(*5) Family Well-Being.* To evaluate family stress associated with their role as firefighters, we asked the following question: My role as a firefighter places stress on my family. Response options were on a scale ranging from strongly disagree, disagree, neither agree or disagree, agree, and strongly agree.

### 2.5. Statistical Approach

Data analysis was conducted with SPSS [57]. The distribution of responses on each CWDH-A item was examined and the proportion of each response category computed. Next, participants' total scores on the CWDH-A scale were used to categorize women firefighters into tertiles of work mistreatment severity. Means ± standard deviation scores or percentages were calculated for all baseline demographic, physical and mental health, substance use, and job efficacy, stress, and satisfaction variables stratified by tertiles of work mistreatment severity.

With respect to the various outcome variables, we dichotomized those that had established cutoffs (e.g., BMI, CESD-10, TSQ, and CAGE). For example, we used national standards to define obesity based on BMI (i.e., ≥30kg/m^2^ [35, 36] and risk for depression, PTSD, and alcohol abuse based on published thresholds for their respective measures, i.e., CESD-10≥4, TSQ≥6, and CAGE≥2 [40, 44, 45]. We also dichotomized some categorical variables whose distributions indicated it, e.g., primarily bimodal distributions, and those where one category was the primary outcome of interest (e.g., current smoker vs. former and never smokers; current smokeless tobacco user vs. former and never users).

We used logistic regression to examine the impact of work discrimination-harassment severity category on dichotomous variables. Nonparametric overall and post hoc tests (Kruskal-Wallis and pairwise comparisons using the Dunn-Bonferroni approach) were used to examine group differences on the MHI-A, FFCSE, and SRPA because of the high degree of skewness in their distributions and the fact that their residuals were not normally distributed, and attempts to transform their distributions failed to normalize them.

Poisson regression [58] was used to explore the association between work discrimination/harassment severity tertile and number of poor physical health days in the last 30 days because they represent count outcomes with distributions that are typically skewed and with zeros represent the modal count (“0”) or no poor physical health days represented 54.4% of distribution. The Poisson model evaluated the effect of work discrimination-harassment severity tertile categories with output that included both *β*-weights and the corresponding odds ratio (OR) for each category of work discrimination-harassment severity and its association with the number of poor physical health days. For all regression models, the referent category for work discrimination-harassment severity was the lowest tertile (the low work discrimination-harassment severity category).

## 3. Results

### 3.1. Participants

A total of 2,022 women career firefighters responded to the survey. The majority were Caucasian (91.9%) and had at least some college degree (96.3%). On average, they had 13.6 years on the job (SD=7.9 years). Of the women who responded, 1,773 (88%) responded to the questions focused on discrimination and harassment.

### 3.2. Work Discrimination and Reports of Harassing Behaviors

**Figure 1:**
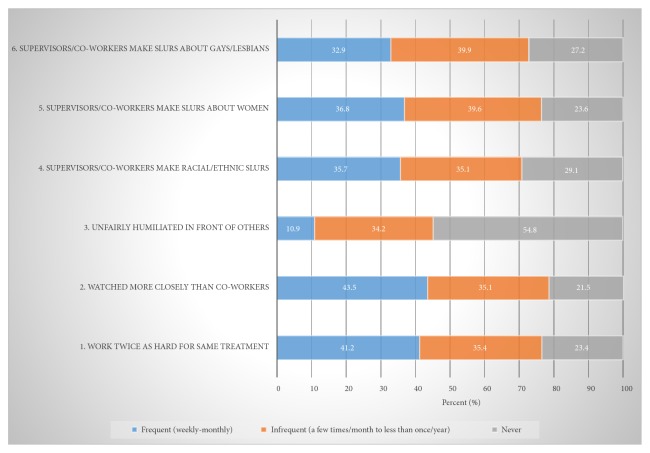
Prevalence of frequent, infrequent, and never categories of job discrimination-harassment items.

The proportions for the different response categories for each of the CWDH-A items are presented in Figure 1.

As can be seen, over 40% of women firefighters reported that they frequently felt that they had to work twice as hard as others to get the same treatment or evaluation and that they were watched more closely than other workers. Over one-third of participants also reported frequently hearing supervisors and/or coworkers making slurs against racial and ethnic minorities, women, and gays and lesbians. Almost 11% reported frequently being humiliated in front of others at work.

**Figure 2:**
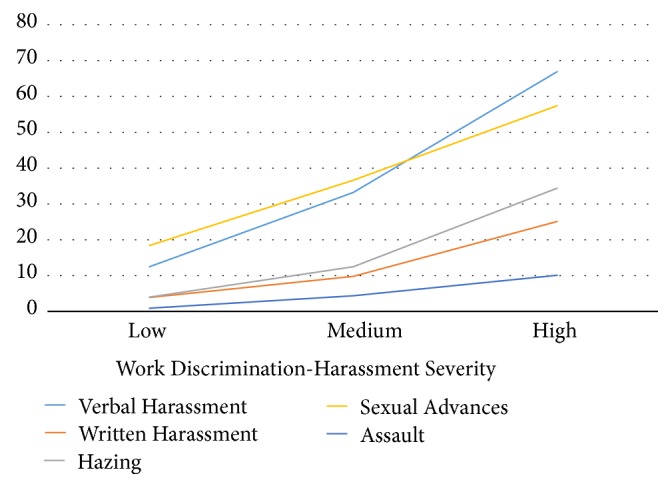
Prevalence and severity of verbal and written harassment, hazing, sexual advances, and assault.

Of the specific types of gender-based harassment, women reported experiencing verbal harassment (37.5%), written harassment (12.9%), hazing (16.9%), sexual advances (37.4%), and assaults (5.1%) because of their gender while in the fire service. When examined in the context of the CWDH-A categories of work discrimination-harassment severity (see Figure 2), women in the highest tertile had significantly greater odds of experiencing each type of harassment when compared to those in the lowest tertile. For example, women in the highest tertile of the CWDH-A severity were 14.2 times (95%CI=10.5-19.1) more likely to experience verbal harassment, 8.3 times (95%CI=5.3-13.1) more likely to report written harassment, 12.4 times (95%CI=8.0-19.3) more likely to experience hazing, 6.0 times (95%CI=4.6-7.8) more likely to experience sexual advances, and 13.1 times (95%CI=5.2-33.0) more likely to report a history of assault compared to those in the lowest tertile (p<0.001 for all contrasts).

As shown in Table 2, even those in the middle tertile (medium severity work discrimination-harassment) had significantly greater odds of reporting the five types of harassment when compared to those in the lowest tertile for verbal harassment (OR=3.5; 95%CI=2.6-4.7), written harassment (OR=2.7; 95%CI=1.6-4.4), hazing (OR=3.4; 95%CI=2.1-5.4), sexual advances (OR=2.6; 95%CI=2.0-3.4), and assaults (OR=5.4; 95%CI=2.0-14.1), compared to those in the lowest tertile (p<0.001 for all contrasts).

### 3.3. Physical Health

There were no significant differences in obesity risk between women firefighters based on the severity of work discrimination-harassment in the last 12 months (see Table 2). In contrast, women firefighters in the highest tertile of work discrimination-harassment severity over the past 12 months reported over 40% more poor health days in the last 30 days (OR=1.42; 95%CI=1.33-1.51; p<0.001) when compared to those in the medium (OR=1.03; 95%CI=0.96-1.10) and low (referent group) categories. The difference between those in the medium and low severity groups was not significant. Odds ratios and confidence intervals are presented in Table 3.

With respect to injury risk, there was a dose-response relationship between the severity of reported work discrimination-harassment and injuries reported in the last year (see Table 2). Women firefighters in the high (OR=2.21; 95%CI=1.75-2.78; <0.001) and medium (OR=1.35; 95%CI=1.07-1.71; p=0.011) severity work discrimination-harassment groups were 120% and 35%, respectively, more likely to report one or more injuries in the previous 12 months when compared to those in low severity category and the difference in risk between the high and medium severity categories also was significant.

### 3.4. Mental Health

Odds of current significant depressive symptoms also were elevated in a dose-response manner based on severity of work discrimination-harassment. Women firefighters in the high severity group were more than 300% (OR=4.20; 95%CI=3.25-5.67; p<0.001) more likely to meet the threshold for significant depressive symptoms when compared to those in the low severity group, while those in the medium severity group were 74% (OR=1.74; 95%CI=1.29-2.34; p<0.001) more likely to meet the threshold. The difference between those in the medium and high group also was significant (see Table 3).

Reported work discrimination-harassment also was significantly associated with current anxiety symptom severity in a dose-response manner (p<0.001 for overall Kruskal-Wallis Test). Post hoc tests revealed that women in the high severity group scored significantly higher on the MHI-A when compared to those in the medium (p<0.001) and low (p<0.001) categories. The medium group also had significantly higher MHI-A scores than those in the low group (p<0.001).

Women firefighters in the high severity work discrimination-harassment group were over 150% (OR=2.67; 95%CI=1.82-3.93; p<0.001) more likely to meet the threshold for potential PTSD when compared to the women in the low severity group. The medium group's 25% greater odds (OR=1.25; 95%CI=0.81-1.93; p=0.314) were not significantly different from the low group and the difference between the medium and high severity groups was not statistically significant.

### 3.5. Health Behaviors (Substance Use, Physical Activity)

Women firefighters in the high (OR=1.54; 95%CI=1.09-2.17; p=0.015) and medium (OR=1.47; 95%CI=1.04-2.07; p=0.029) work discrimination-harassment severity groups were significantly more likely to demonstrate elevated odds for meeting the CAGE threshold for alcohol abuse when compared to those in the low severity category. However, there was no statistical difference between those in the high and medium groups (see Table 2).

Women in the medium severity group for work discrimination-harassment were nearly three times (OR=2.71; 95%CI=1.33-5.50; p=0.006) more likely to report having driven while intoxicated in the last 30 days when compared to those in the low group. While elevated risk also was evident in the high severity group (OR=2.10; 95%CI=0.98-4.32), it was not statistically different from the low or medium groups. There were no significant group differences based on severity of work discrimination-harassment on binge drinking, smoking, or smokeless tobacco use.

### 3.6. Job Efficacy/Stress/Satisfaction

There was an inverse relationship between women firefighters' confidence in managing on-the-job stressful and traumatic experiences, as measured by the FFCSE (p<0.001 for overall Kruskal-Wallis Test). Post hoc analysis revealed that women firefighters in the high (p<0.001) and medium (p<0.001) severity work discrimination-harassment groups had significantly lower scores on the FFCSE than those in the low severity group. However, the difference between the high and medium severity groups was not statistically significant.

Women firefighters who reported the most severe work discrimination-harassment also reported the most work-related stress and that stress interfered with their ability to carry out their firefighter duties over the last year. Women in the high severity work discrimination group were more than 200% (OR=3.36; 95%CI=2.57-4.40; p<0.001) and nearly 600% (OR=6.79; 95%CI=2.85-16.19; p<0.001) more likely to select the highest categories (“a lot” in the last 12 months) of work-related stress and stress interfering with their ability to do their jobs when compared to those in the low severity group.

Women firefighters in the medium severity group also were significantly more likely (OR=1.52; 95%CI=1.14-2.03; p=0.004) to report “a lot” of work stress in the past 12 months and the difference between those in the high and medium groups also was significantly different on the stress item. There was no significant difference between women firefighters in the medium (OR=1.74; 95%CI=0.63-4.83; p=0.285) and low work discrimination-harassment severity groups on the stress interference item, nor was the difference between high and medium work discrimination groups risk significantly different.

With respect to the five job satisfaction items, four (i.e., optimism about future; satisfaction with job; recommend being a firefighter, and happy to spend rest of career as a firefighter) demonstrated the same inverse dose-response patterns, with those in the high severity work discrimination-harassment group being significantly less likely to report they agreed or strongly agreed that they were optimistic about their future (OR=0.23; 95%CI=0.18-0.30; p<0.001), were satisfied with their job in the department (OR=0.18; 95%CI=0.13-0.25; p<0.001), would recommend being a firefighter to other women (OR=0.31; 95%CI=0.24-0.41; p<0.001), and would be happy to spend the rest of their career with their fire department (OR=0.19; 95%CI=0.14-0.25; p<0.001) when compared to the low severity group.

Those in the medium severity group for work discrimination-harassment also were significantly less likely to agree or strongly agree with the above four items (OR=0.55; 95%CI=0.41-0.72; p<0.001 for optimism; OR=0.48; 95%CI=0.35-0.67; p<0.001 for job satisfaction; OR=0.57; 95%CI=0.43-0.75; p<0.001 for recommending job to other women; and OR=0.43; 95%CI=0.31-0.60; p<0.001 for being happy to spend the rest of their career in their fire department). The differences between the high and medium work discrimination-harassment severity groups also were significant.

On the item about their happiness with their choice of being a firefighter, both the high (OR=0.38; 95%CI=0.26-0.57; p<0.001) and medium (OR=0.65; 95%CI=0.42-0.99; p<0.047) work discrimination-harassment severity groups were significantly less likely to agree or strongly agree when compared to the low severity group, but the difference between those in the high and low groups was not significant.

### 3.7. Family Well-Being

Women firefighters in the highest and middle tertiles of work discrimination-harassment were nearly three times (OR=2.91; 95%CI=2.30-3.70; p<0.001) and 69% (OR=1.69; 95%CI=1.33-2.15; p<0.001) more likely agree or strongly agree that their role as a firefighter placed stress on their family. In addition, OR for those in the high severity work discrimination-harassment was significantly higher than that found for women firefighters in the middle tertile.

## 4. Discussion

An alarming number of women reported experiencing gender-based harassment while at work. More than a third reported verbal harassment and a similar number reported sexual advances. Slightly more than 5% reported experiencing assaults. These results are similar to those in military populations where 27% of women report unwanted sexual attention and 8% of women report sexual coercion [59].

Our data show that women firefighters in the medium and high tertiles of chronic work discrimination-harassment on the CWDH-A were significantly more likely to report incidents of verbal and written harassment, hazing, sexual advances, and assault than women in the lowest tertiles, demonstrating disturbingly high rates of these incidents in a mostly dose-response fashion on discrimination-harassment severity gradient of CWDH-A tertiles. In addition, it also provides data on the excellent concurrent validity of the CWDH-A and the work discrimination-harassment severity categories created from it for the subsequent analyses.

Consistently, women who experienced moderate and severe discrimination and harassment had negative mental health outcomes including higher prevalence of depressive symptoms, anxiety, and symptoms of PTSD. Those women who had experienced high rates of discrimination and harassment also were at high risk for potential issues with alcohol consumption as measured by the CAGE questions. The highest severity group also was least likely to be satisfied with their job or recommend being a firefighter to others which lends credence to the suggestion that discrimination and harassment lead to recruitment and retention issues [13].

The impact of discrimination and harassment, the related negative physical and mental outcomes, the low levels of job satisfaction, and the negative impact of these experiences on family/home stress likely take a significant toll on women in the fire service. While the current study did not examine suicidal ideation directly, examination of death certificates indicates that women in the protective services (law enforcement and fire service) have the highest rates of suicide of any of the professions studied [60]. The high rates of discrimination and harassment reported and the negative outcomes related to these experiences are potential explanations for these rates.

Discrimination and harassment also were related to injuries in the past 12 months. Given the cross-sectional nature of the data, it is unclear whether the injuries increased risk for discrimination and harassment or vice versa. However, data from Hollerbach and colleagues [61] suggests that women may push themselves harder than necessary and put themselves in unsafe settings to “prove” themselves to their male colleagues. It is plausible that these efforts put women who experience chronic discrimination and harassment at higher risk.

The current study is the largest to date of career women firefighters examining the impact of chronic work discrimination-harassment on health status. Considering that there are approximately 350,000 career firefighters in the US and that women represent between 3.5%-5.1% (≈12,250-17,850 [12, 13, 62]), our study likely represents 9.9%-14.5% of all women firefighters in the US, with representation from all but one US state. Thus, while not a random sample, our sample and the resulting data are the largest and most representative sample of career women firefighters to date.

## 5. Study Limitations

The primary limitations of this study are the sampling method and the potential for response bias, the fact that the parent study from which these data are drawn had very different goals, the cross-sectional design, and the fact that all physical and mental health and job-related outcomes were based on self-report. As we noted earlier, there is no logistically feasible method enumerating the sampling frame for women firefighters in the US (i.e., there are no national lists of firefighters in the US and departments typically are reluctant to release personnel data). Thus, the sampling approach used in this study represented the most reasonable strategy to reach a large number of women firefighters. And as we noted previously, our sample size represents a large proportion of the total estimated number of career women firefighters in the US.

The parent study was designed as a mixed-methods investigation that included an epidemiological survey of women firefighters examining a broad range of health concerns, with a particular focus on reproductive health concerns. The focus on reproductive health was justified because anecdotal data and a few limited studies indicated that women firefighters have concerns about the impact of firefighting job tasks on their reproductive health, which ultimately may impact recruitment and retention of women firefighters [12]. However, that focus limited our ability to thoroughly examine other potentially important domains relevant to this paper. Nevertheless, we used a well-known and validated measure of chronic work discrimination-harassment, as well as strong measures for the other health domains. It should be noted that the harassment measured by the CWDH-A focuses on harassment in the work environment but does not ask specifically about personal experiences with harassment. Future research should ask specifically about personal experiences.

Also, because the study was cross-sectional, it is not possible to determine the direction of relationships between work discrimination-harassment and health outcomes. Future work should explore prospective relationships between discrimination, harassment, and the effect on health outcomes. All outcome measures were based on self-report. However, measures such as self-reported weight and substance use have been found to be highly correlated with objectively measured outcomes [29, 45, 63]. The study also focused solely on women so direct comparisons to the men in the same settings was not possible. Further, the survey relied on valid and reliable measures of health status and experience. In addition, the number of poor physical health days is used as an index of health-related quality of life and has demonstrated predictive validity with both health care utilization and mortality [33, 34].

## 6. Implications

Findings confirm and extend previous work on this topic that suggests there is a considerable amount of work to be done to improve the mental and physical health of women firefighters. For instance, behavioral health programs and health/wellness programs in the fire service should highlight the increased risks women experience related to discrimination and harassment. Education about the impact of this workplace mistreatment should be included as part of the behavioral health trainings. It is likely that discrimination and harassment of women, a relative minority in the fire service, undermine their access to the main protective benefit of the camaraderie and bonding so common in the fire house [64]. Future work should also examine the prospective relationship between discrimination and harassment and poor health outcomes as well as potential policies and practices that can reduce these negative behaviors. Examining the intersectionality of gender, race, and gender identity will also likely prove useful among this population [65].

Overall, this study highlights the high rates of both perceived discrimination and harassment among female firefighters. While rates of behavioral health concerns among females in the fire service tend to be higher than rates in the published literature for male firefighters [66, 67], the current study suggests that this relationship might be due to experienced or perceived discrimination or harassment. In fact, when examining those with limited experiences of discrimination or harassment, rates of behavioral health concerns closely mirrored those evidenced among male firefighters. It is possible that being singled out and receiving negative differential treatment not only has a negative impact on behavioral health directly but also robs firefighters being discriminated against or harassed of the camaraderie that has been found to be so protective for firefighters [64]. In addition, while the current study did not specifically examine their impact on recruitment and retention, findings do have implications for what fire departments need to consider to effectively recruit and retain a more diverse workforce. It is clear that departments need to be vigilant about preventing and addressing discrimination and harassment both with policy and actions as they clearly lead to poor outcomes among women in the fire service. Allowing these issues to go unchecked could logically lead to women choosing to leave the fire service.

## Figures and Tables

**Table 1:** Participant Demographics.

Characteristic	Mean ± SD; %
Age (years; M ± SD)	40.2 ± 9.0
Race (%)	
(i) White	91.9%
(ii) African American/Black	3.7%
(iii) Asian American	0.8%
(iv) Native Hawaiian/Pacific Islander	0.4%
(v) American Indian/Alaskan Native	0.9%
(vi) Other	2.6%
Hispanic Origin (% yes)	6.4%
Marital Status (% Married/Domestic Partner, Civil Union)	55.5%
Sexual Orientation (%)	
(i) Heterosexual/Straight	79.4%
(ii) Lesbian	14.6%
(iii) Bisexual	4.0%
(iv) Other	0.2%
(vi) Refused to Answer	1.8%
Education (% at least some college)	96.3%
Annual Household Income (%)	
(i) <$50k	9.7%
(ii) ≥$50k	45.3%
Rank (%)	
(i) Firefighter, Firefighter/Medic, Medic, Driver Operator	69.6%
(ii) Company Officer (Lieutenant, Captain)	24.3%
(iii) Any Chief (Battalion Chief, Assistant Chief, Deputy Chief, Chief)	6.1%
Fire Service Experience (years; M ± SD)	13.6 ± 7.9

**Table 2:** Outcomes by Discrimination Severity.

Variable	*Tertiles of Harassment-Discrimination Severity*			p-value
	Low	Medium	High	
	n=603	n=578	n=592	
*Demographic Characteristics *				
Age (years; M ± SD)	40.3 ± 9.2	40.4 ± 8.9	39.8 ± 8.9	0.452
Race (% White, Non-Hispanic)	86.3	88.7	86.6	0.420
Marital Status (% Married, domestic partnership, or civil union)	56.0	58.7	51.8	0.059
Sexual Orientation (% Heterosexual)	79.8	81.3	77.1	0.204
Education (% At least some college or higher)	95.0	97.2	96.7	0.117
Income (% $50,000 or more)	91.1	91.5	88.4	0.146

*Occupational Characteristics*				
Years in the Fire Service (years; M ± SD)	13.4 ± 8.1	13.7 ± 8.0	13.7 ± 7.6	0.783
Rank (% Any firefighter rank)	71.5	67.6	69.8	0.452

*(1) Physical Health*				
Obesity (% BMI≥30 kg/m^2^)	13.5	14.5	12.6	0.653
Number of Poor Physical Health Days (M ± SD)^*¥*^	2.8 ± 6.7	2.9 ± 6.7	4.0 ± 7.3	<0.001
Injuries Reported in Last Year (% 1 or more)^*∗*^	36.8	44.1	56.3	<0.001

*(2) Mental Health*				
Current Depression (CESD-10 Depression Cutoff; % ≥4)^*∗*^	15.1	23.6	43.3	<0.001
Current Anxiety Symptoms (MHI Anxiety Score Total; M ± SD)^*∗∗*^	16.4 ± 6.5	17.7 ± 5.9	21.1 ± 7.6	<0.001
Current PTSD Symptoms (TSQ Cutoff; %≥6)^*∗*^	6.9	8.5	16.5	<0.001

*(3) Health Behaviors (Substance Use, Physical Activity)*				
CAGE Cutoff (%≥2)^*∗*^	13.4	18.6	19.2	0.027
Binge Drinker (% yes)	45.5	52.9	47.4	0.056
Drove While Intoxicated (% yes)^*∗*^	2.2	5.7	4.4	0.014
Smoke Cigarettes (% current)	4.0	5.9	4.8	0.120
Smokeless Tobacco Use (% current)	1.5	1.2	1.2	0.846
Physical Activity Level (SRPA; M ± SD)	5.7 ± 1.4	5.5 ± 1.5	5.5 ± 1.5	0.787

*(4) Job Outcomes (Efficacy/Stress/Satisfaction)*				
FFSE Score Total (M ± SD)^*∗∗*^	118.8 ± 14.3	115.4 ± 12.3	113.8 ± 13.0	<0.001
How Much Stress at Work (% a lot)^*∗*^	16.7	23.4	40.3	<0.001
Stress Interferes With Work (% a lot)^*∗*^	1.0	1.7	6.4	<0.001
Optimistic about future success in fire department (% agree/strongly agree)^*∗*^	82.1	71.4	51.5	<0.001
Satisfied with job in fire department (% agree/strongly agree)^*∗*^	89.2	80.0	59.9	<0.001
Happy with choice to be a firefighter (% agree/strongly agree)^*∗*^	93.4	90.1	84.3	<0.001
Would recommend being a firefighter to other women (% agree/strongly agree)^*∗*^	82.6	72.9	59.7	<0.001
Happy to spend the rest of career with fire department (% agree/strongly agree)^*∗*^	88.7	77.3	59.3	<0.001

*(5) Family Well-Being *				
Role as a Firefighter Places Stress on Family (% agree/strongly agree)^*∗*^	24.0	32.3	55.1	<0.001

Note: ^*∗*^Dichotomous outcomes were modeled using logistic regression. ^*∗∗*^The MHI-A, FFSE, and SRPA were modeled using nonparametric overall and post-hoc tests. ^*¥*^Number of poor physical health days in the last 30 was modeled with Poisson regression.

**Table 3:** Odds Ratios and Confidence Intervals.

	Work Discrimination & Harassment Severity (CWDH-A)		
	Low Severity	Medium Severity	High Severity
	(Referent Group)		
Variable		OR (CI)^a^	OR (CI)^a^
Discrimination & Harassment^b^			
Verbal Harassment	-	3.49 (2.58 to 4.70)^*∗*^	14.20 (10.54 to 19.13)^*∗*^
Written Harassment	-	2.70 (1.64 to 4.44)^*∗*^	8.30 (5.26 to 13.10)^*∗*^
Hazing	-	3.37 (2.09 to 5.44)^*∗*^	12.41 (7.97 to 19.32)^*∗*^
Sexual Advances	-	2.57 (1.97 to 3.37)^*∗*^	6.00 (4.61 to 7.82)^*∗*^
Assault	-	5.38 (2.04 to 14.15)^*∗*^	13.13 (5.23 to 32.96)^*∗Ɨ*^

Physical Health			
Poor Health Days^c^	-	1.03 (0.96 to 1.10)	1.42 (1.33 to 1.51)^*∗*^
Injury Risk^b^	-	1.35 (1.07 to 1.71)^*∗*^	2.21 (1.75 to 2.78)^*∗*^

Mental Health^b^			
Depression	-	1.74 (1.29 to 2.34)^*∗*^	4.20 (3.25 to 5.67)^*∗*^
PTSD	-	1.25 (0.81 to 1.93)	2.67 (1.82 to 3.93)^*∗Ɨ*^

Health Behaviors^b^			
Alcohol (CAGE)	-	1.47 (1.04 to 2.07)^∧^	1.54 (1.09 to 2.17)^∧*Ɨ*^
Driving Intoxicated	-	2.71 (1.33 to 5.50)^∧^	2.10 (0.98 to 4.32)^*Ɨ*^

Job Outcomes^b^			
Work-related stress	-	1.52 (1.14 to 2.03)^∧^	3.36 (2.57 to 4.40)^*∗*^
Stress-Interfering	-	1.74 (0.63 to 4.83)	6.79 (2.85 to 16.19)^*∗Ɨ*^
Optimism	-	0.55 (0.41 to 0.72)^*∗*^	0.23 (0.18 to 0.30)^*∗*^
Satisfaction	-	0.48 (0.35 to 0.67)^*∗*^	0.18 (0.13 to 0.25)^*∗*^
Recommend FF	-	0.57 (0.43 to 0.75)^*∗*^	0.31 (0.24 to 0.41)^*∗*^
Happy with Career	-	0.43 (0.31 to 0.60)^*∗*^	0.19 (0.14 to 0.25)^*∗*^
Happy rest of career	-	0.65 (0.42 to 0.99)^∧^	0.38 (0.26 to 0.57)^*∗Ɨ*^

Family Well-Being^b^			
Stress on Family	-	1.69 (1.33 to 2.15)^*∗*^	2.91 (2.30 to 3.70)^*∗*^

Note: ^a^Odds Ratios given with confidence interval. ^b^Logistic Regression, ^c^Poisson Model. ^*∗*^Statistically significant (p<0.001); ^∧^Statistically significant (p<0.05); ^*Ɨ*^Statistically significant difference between medium and high severity groups.

## Data Availability

The deidentified dataset used to support the findings of this study are available from the corresponding author upon request and completion of a data sharing agreement and the approval of the NDRI signing official.
